# Self-attenuation of extreme events in Navier–Stokes turbulence

**DOI:** 10.1038/s41467-020-19530-1

**Published:** 2020-11-17

**Authors:** Dhawal Buaria, Alain Pumir, Eberhard Bodenschatz

**Affiliations:** 1 Max Planck Institute for Dynamics and Self-Organization, Göttingen, 37077 Germany; 2 Tandon School of Engineering, New York University, New York, 11201 USA; 3 Laboratoire de Physique, Ecole Normale Supérieure de Lyon, Université de Lyon 1 and Centre National de la Recherche Scientifique, Lyon, 69007 France; 4 Institute for Nonlinear Dynamics, University of Göttingen, G, 37077 Germany

**Keywords:** Computational science, Fluid dynamics, Statistical physics, thermodynamics and nonlinear dynamics

## Abstract

Turbulent fluid flows are ubiquitous in nature and technology, and are mathematically described by the incompressible Navier-Stokes equations. A hallmark of turbulence is spontaneous generation of intense whirls, resulting from amplification of the fluid rotation-rate (vorticity) by its deformation-rate (strain). This interaction, encoded in the non-linearity of Navier-Stokes equations, is non-local, i.e., depends on the entire state of the flow, constituting a serious hindrance in turbulence theory and even establishing regularity of the equations. Here, we unveil a novel aspect of this interaction, by separating strain into local and non-local contributions utilizing the Biot-Savart integral of vorticity in a sphere of radius *R*. Analyzing highly-resolved numerical turbulent solutions to Navier-Stokes equations, we find that when vorticity becomes very large, the local strain over small *R* surprisingly counteracts further amplification. This uncovered self-attenuation mechanism is further shown to be connected to local Beltramization of the flow, and could provide a direction in establishing the regularity of Navier-Stokes equations.

## Abstract (web summary)

Whether a turbulent flow would inevitably develop singular behavior at the smallest length scales is an ongoing intriguing debate. Using large-scale numerical simulations, Buaria et al. find an unexpected non-linear mechanism which counteracts local vorticity growth instead of enabling it.

## Introduction

A parcel of fluid moving at velocity **u**(**x**, *t*) in a flow, where ${\bf{x}}\in {{\mathbb{R}}}^{3}$ is the spatial location and *t* is time, simultaneously undergoes rotation and shape deformation, respectively, characterized by the vorticity vector ***ω*** = ∇ × **u** and the strain rate tensor *S*_*ij*_ = (∂_*j*_*u*_*i*_ + ∂_*i*_*u*_*j*_)/2. Its evolution in time can thereby be described by the incompressible Navier–Stokes equations (INSE) written as the vorticity equation^1^:

1 $${D}_{t}{\omega }_{i}={\omega }_{j}{S}_{ij}+\nu {\nabla }^{2}{\omega }_{i}\,,$$

where *D*_*t*_ = ∂_*t*_ + *u*_*j*_∂_*j*_ is the material derivative and *ν* is the kinematic viscosity of the fluid. This equation simply expresses that along a parcel trajectory vorticity is non-linearly stretched by the strain rate, and also subjected to viscous damping. An essential aspect of this stretching term is that it causes amplification of vorticity, i.e., generation of enstrophy Ω = *ω*_*i*_*ω*_*i*_, via the production term *P*_Ω_ = *ω*_*i*_*ω*_*j*_*S*_*ij*_, as readily seen by taking the dot product of Eq. (1) with *ω*_*i*_^2^. The rate at which enstrophy is amplified, and whether it can overcome viscous damping to blow-up in finite time, remains one of the outstanding unsolved Clay Millennium Prize problems^3,4^.

It is known that for a finite-time blow-up, *P*_Ω_ must grow unbounded^5^. In addition, it has also been proven that this unbounded growth can possibly only occur when the viscosity *ν* is sufficiently small^4^, which would correspond to turbulent solutions of the INSE. In fact, it is well known that Ω is highly intermittent in turbulent flows, attaining values hundreds or thousands times its mean, becoming even more extreme as the relative strength of viscosity is decreased^6–12^. However, these extreme events are typically found to be arranged in tube-like structures^6–12^, with geometrical properties deterring maximum possible amplification^2,13–15^. Nevertheless, the question remains open whether the non-linear amplification could overcome viscous damping when the flow is sufficiently turbulent.

A fundamental difficulty in analyzing Eq. (1) arises from the non-local coupling between vorticity and strain rate; which implies that strain acting on vorticity at a point, as in Eq. (1), is in fact coupled to the entire state of the flow. Specifically, this non-locality can be quantified by expressing the strain tensor as a Biot-Savart integral of the vorticity field over the entire 3D spatial domain:

2 $${S}_{ij}({\bf{x}})=PV\int_{{{\bf{x}}}^{\prime}}\frac{3}{8\pi }({\epsilon }_{ikl}{r}_{j}+{\epsilon }_{jkl}{r}_{i}){\omega }_{l}({{\bf{x}}}^{\prime})\,\frac{{r}_{k}}{{r}^{5}}\,{d}^{3}{{\bf{x}}}^{\prime}\,,$$

where ${\bf{r}}={\bf{x}}-{{\bf{x}}}^{\prime}$ (with *r* = ∣**r**∣) and *ϵ*_*ijk*_ is the alternating Levi-Civita symbol. Thus, the amplification of vorticity can be entirely written in terms of vorticity itself, but the above integral poses a serious mathematical challenge in understanding the mechanisms encoded in the non-linearity. In the current work, by evaluating the above integral numerically, we provide evidence that as vorticity is amplified to large values, the strain induced locally will ultimately act to attenuate its further amplification.

In order to extract the local strain induced from vorticity amplification, we consider the following decomposition, by splitting the integration domain into a spherical neighborhood of radius *R* and the remaining domain^16,17^:

3 $${S}_{ij}({\bf{x}})=\underbrace{{\int}_{r \,{> }\,R}\left[\cdot \cdot \cdot \right]{d}^{3}{\bf{x}}^{\prime} }_{ = {S}_{ij}^{{\rm{NL}}}({\bf{x}},R)}+\underbrace{{\int}_{r\le R}\left[\cdot \cdot \cdot \right]{d}^{3}{\bf{x}}^{\prime} }_{ = {S}_{ij}^{{\rm{L}}}({\bf{x}},R)}\,,$$

where $\left[\cdots \ \right]$ denotes the integrand in Eq. (2). The first term ${S}_{ij}^{{\rm{NL}}}$ is the non-local or background strain acting on the vorticity to stretch it, whereas ${S}_{ij}^{{\rm{L}}}$ is the local strain, induced by the vorticity in its neighborhood in response to the stretching. Thereafter, the production term can also be decomposed as ${P}_{\Omega }={P}_{\Omega }^{{\rm{L}}}+{P}_{\Omega }^{{\rm{NL}}}$, where ${P}_{\Omega }^{{\rm{L}},{\rm{NL}}}={\omega }_{i}{\omega }_{j}{S}_{ij}^{{\rm{L}},{\rm{NL}}}$. For such a decomposition, explicit bounds on ${P}_{\Omega }^{{\rm{NL}}}$ can be established in terms of the total kinetic energy of the flow^18^. Thus, an unbounded growth of *P*_Ω_ is only possible through ${P}_{\Omega }^{{\rm{L}}}$.

However, in this paper, our results will demonstrate, that when *R* is small enough, the term ${P}_{\Omega }^{{\rm{L}}}$ remarkably acts to attenuate extreme vorticity fluctuations. Further analysis reveals that this attenuation is also connected to local Beltramization of the flow, i.e., preferential alignment of vorticity with velocity, which is expected to deplete the growth of non-linearity^19^.

## Results

### Direct numerical simulations

To analyze the complex interaction between strain and vorticity, we utilize our unique database generated through direct numerical simulations (DNS) of the INSE. The simulations correspond to canonical setup of forced homogeneous and isotropic turbulence in a periodic domain^11^, and are performed using the well-known Fourier pseudo-spectral methods, thus allowing us to obtain any quantity of interest with highest accuracy practicable^20^. It is instructive to note that the mathematical results typically obtained in ${{\mathbb{R}}}^{3}$ can be readily generalized to our simulation in the ${{\mathbb{T}}}^{3}$ torus. Using the largest grid sizes currently feasible in turbulence simulations, of up to 12288^3^ points^21,22^, the Taylor-scale Reynolds number *R*_*λ*_, which quantifies the turbulence intensity, is varied from 140 to 1300 in our simulations (corresponding to fully developed turbulence). Special attention is given to faithfully resolve the small scales and hence the extreme events^12^, keeping the grid spacing smaller than the Kolmogorov length scale, $\eta ={({\nu }^{3}/\langle \epsilon \rangle )}^{1/4}$, based on the mean dissipation rate of kinetic energy 〈*ϵ*〉, where the average 〈 ⋅ 〉 is taken over the 3D spatial domain and also multiple realizations. Note that the mean enstrophy 〈Ω〉, is equal to 〈*ϵ*〉/*ν*, owing to underlying homogeneity^1^. Additional details about our DNS and database are provided in the Methods section.

### Robust determination of the local and non-local strain

Although the vorticity and strain fields can be easily obtained from DNS, we have devised an efficient method to compute the local and non-local strain fields, without directly evaluating the prohibitively expensive Biot-Savart integral over the entire domain. As shown in ref. ^16^, using a Taylor-series expansion of vorticity over a distance *R*, the non-local strain **S**^NL^(**x**, *R*) can be expressed in terms of the total strain as follows:

4 $${S}_{ij}^{{\rm{NL}}}({\bf{x}},R)=	 \left[1+\frac{{R}^{2}}{10}{\nabla }^{2}+\frac{{R}^{4}}{280}{\nabla }^{2}{\nabla }^{2}+\ldots \right.\\ 	\left.+\frac{3{R}^{2n-2}}{(2n-2)!(4{n}^{2}-1)}{({\nabla }^{2})}^{n-1}+...\right]{S}_{ij}({\bf{x}}).$$

Starting from the above expression and transforming it to Fourier space (where the differential operator ∇^2^ reduces to a simple multiplication by  −*k*^2^), leads to the relation

5 $${\hat{S}}_{ij}^{{\rm{NL}}}({\bf{k}},R)=f(kR){\hat{S}}_{ij}({\bf{k}})\,,$$

where $({\hat{\cdot}})$ denotes the Fourier transform, **k** is the wavenumber vector with *k* = ∣**k**∣ and *f*(*kR*) is an infinite series. In practice, truncating *f*(*kR*) to a finite number of terms can at best provide approximate results^16^. However, as derived in the Supplementary Note 1, one can show that *f*(*kR*) converges to the following expression:

6 $$f(kR)=\frac{3\left[\sin (kR)-kR\cos (kR)\right]}{{(kR)}^{3}}\,.$$

This allows us to evaluate the Biot-Savart integral in Eq. (2) by applying a simple transfer function to the total strain rate in Fourier space, and thus to obtain ${S}_{ij}^{{\rm{L}},{\rm{NL}}}$ (and ${P}_{\Omega }^{{\rm{L}},{\rm{NL}}}$) very accurately for any value of *R*. Interestingly, it is worth noting that *f*(*kR*) in Eq. (6) corresponds to the sinc function in 3D, which also happens to be the Fourier transform of a box or top-hat filter (of radius *R*), commonly utilized in other disciplines, e.g., large-eddy simulation, signal processing. Thus, evaluating the non-local strain seemingly amounts to a filtering operation on the total strain.

### Visualization of extreme events

**Fig. 1:**
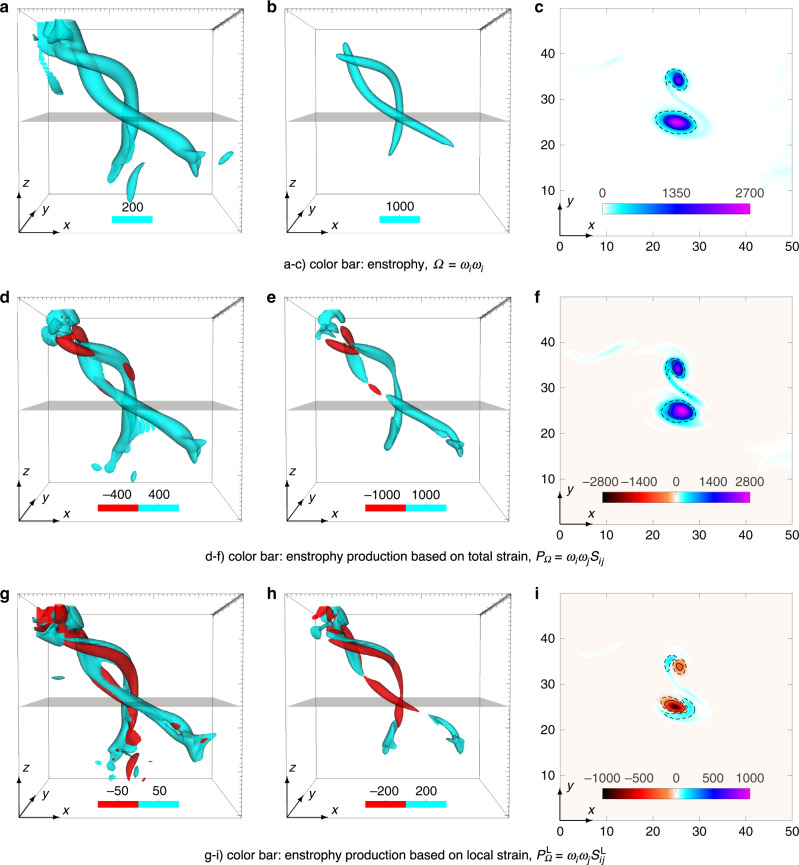
Prevalence of negative local stretching in regions of intense vorticity. The panels focus on a representative region of intense vorticity from the numerical simulation at Taylor-scale Reynolds number *R*_*λ*_ = 650 on a 8192^3^ grid or equivalently (4096*η*)^3^, where *η* is the Kolmogorov length scale. The maximum enstrophy (vorticity-squared) is at the center of the domain shown, whose edges are 50*η* in each direction (in each panel successive major ticks are 10*η* apart). Top row: isosurfaces of enstrophy at thresholds of **a** 200, and **b** 1000 (times the mean value). **c** 2D contours of enstrophy at the mid-plane of the domain, shown in gray in **a** and **b**. Middle row: enstrophy production based on total strain, suitably non-dimensionalized by mean of enstrophy, at thresholds of **d**  ±400, and **e**  ±1000, which approximately correspond to moderate and intense enstrophy, shown in **a** and **b**, respectively. **f** 2D contours at the mid-plane. The production terms based on total strain is overwhelmingly positive. Bottom row: enstrophy production based on local strain (for *R* = 2*η*), once again suitably non-dimensionalized by mean enstrophy, at thresholds of **h**  ±50, and **g**  ±200, and also corresponding to moderate and intense enstrophy shown in **a**, **b**, respectively. **i** 2D contours at the mid-plane, revealing that the production term based on local strain is strongly negative in the regions of intense vorticity. For each row, the thresholds shown in first two isosurfaces plots are marked by dashed and solid lines respectively in last 2D contour field plot.

  Figure. 1 illustrates our main result, namely that the local contribution to stretching, ${P}_{\Omega }^{{\rm{L}}}$, is in fact negative in the neighborhood of extreme vorticity events. The visualizations shown in Fig. 1 focus on a small domain of size (50*η*)^3^ around one of the extreme vorticity events in the flow (with the most intense vorticity at the center). Figure. 1a, b show isosurfaces of enstrophy, respectively, at 100 and 1000 times the mean value corresponding to moderate and intense events, and illustrate the characteristic vortex tube structure^10–12^. The cut through the mid-plane of the domain is shown in Fig. 1c, and demonstrates the sharp variation of enstrophy across the cross section of the tubes.

  Figure. 1d–f show the total production *P*_Ω_ for the same field. In Fig. 1d, isosurfaces are shown for levels  ±400 (with cyan and red corresponding to positive and negative values, respectively), which approximately correspond to moderate enstrophy (as shown in Fig. 1a). Whereas in Fig. 1e, isosurfaces are shown for  ±1000, which correspond to intense enstrophy (as shown in Fig. 1b). In Fig. 1f, the 2D contour field at the mid-plane is shown. The main observation is that *P*_Ω_ is overwhelmingly positive, which is anticipated given large enstrophy in these tubes, and also from dynamical constraints of turbulence^23^.

Finally, Fig. 1g–i shows the contribution ${P}_{\Omega }^{{\rm{L}}}$ from local strain for *R* = 2*η*. In Fig. 1g and h, isosurfaces are shown for levels  ±50 and  ±200, respectively, once again corresponding to moderate and intense enstrophy events, respectively. Unlike *P*_Ω_ that is always positive on average^23^, the mean of ${P}_{\Omega }^{{\rm{L}}}$ has no such constraints. For moderate values shown in Fig. 1g, we find that the volumes occupied by positive and negative values are comparable. However, for intense value shown in Fig. 1h, negative stretching rate is more prevalent especially around the center where vorticity is maximum. This is corroborated by Fig. 1i, which shows the 2D contour level of ${P}_{\Omega }^{{\rm{L}}}$ at the mid plane and reveals that both negative and positive values occur in the outer regions of the tubes where vorticity is not very intense; whereas large negative values occur inside the tubes, where vorticity is most intense.

Let us briefly mention that the flow structure presented in Fig. 1 represents one generic scenario of how the regions of intense vorticity look like. Needless to say, we inspected many such regions, and note that all of them qualitatively behave in the same manner, and essentially lead to the same conclusion. We have shown another such example in the Supplementary Fig. 1.

### Conditional statistics

**Fig. 2:**
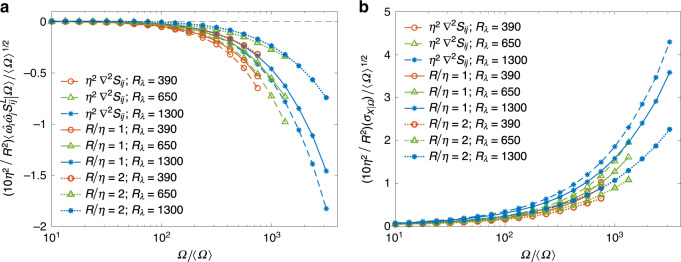
Negative contribution of local strain to production of enstrophy. **a** Averaged enstrophy production due to the local strain, ${P}_{\Omega }^{{\rm{L}}}$, conditioned on enstrophy normalized by its mean value. The curves shown correspond for *R*/*η* = 1 and 2, at Taylor-scale Reynolds numbers *R*_*λ*_ = 390–1300. For comparison, we also show the contribution based on *η*^2^∇^2^*S*_*ij*_, which is the limiting value of local strain for small *R* as noted in Eq. (7). (Accordingly the curves for *R*/*η* = 1 and 2 are also adjusted by a factor of 10*η*^2^/*R*^2^). **b** The conditional root-mean-square *σ*_*X*∣Ω_ of the local enstrophy production term ($X={\hat{\omega }}_{i}{\hat{\omega }}_{j}{S}_{ij}^{{\rm{L}}}$), defined as ${\sigma }_{X| \Omega }^{2}=\langle {X}^{2}| \Omega \rangle -{\langle X| \Omega \rangle }^{2}$. Similar normalization as **a** is used.

To establish the quantitative significance of the observations in Fig. 1, Fig. 2a shows the average of ${P}_{\Omega }^{{\rm{L}}}/\Omega$ conditioned on Ω, for *R* = *η* and 2*η*, and various Reynolds numbers. Note ${P}_{\Omega }^{{\rm{L}}}/\Omega =\hat{{\omega }_{i}}\hat{{\omega }_{j}}{S}_{ij}^{{\rm{L}}}$ (where $({\hat{\cdot}})$ is the corresponding unit vector) and provides the measure of effective strain engendering enstrophy production, irrespective of the strength of vorticity^2,13^. The Taylor expansion in Eq. (4) implies that for small *R*, ${S}_{ij}^{{\rm{L}}}$ can be written as

7 $${S}_{ij}^{{\rm{L}}}({\bf{x}},R)=-\frac{{R}^{2}}{10}{\nabla }^{2}{S}_{ij}({\bf{x}})+{\mathcal{O}}({R}^{4})\,,$$

which suggests that the local strain is in fact proportional to the Laplacian of the total strain. Hence for comparison, we have also shown the conditional expectation $\langle {\hat{\omega }}_{i}{\hat{\omega }}_{j}{\eta }^{2}{\nabla }^{2}{S}_{ij}| \Omega \rangle$ in Fig. 2a, and ${P}_{\Omega }^{{\rm{L}}}$ is accordingly multiplied by 10*η*^2^/*R*^2^. The conditional production term is virtually zero for small to moderate values of Ω—consistent with strong cancellation between negative and positive values seen in Fig. 1g. However, as Ω gets larger, the expectation $\langle {P}_{\Omega }^{{\rm{L}}}| \Omega \rangle$ becomes negative for all Reynolds numbers and strongly increases in magnitude with Ω. We note that the values of ${P}_{\Omega }^{{\rm{L}}}$ are overwhelmingly negative for large Ω, as corroborated by the observation (not shown in figure) that conditional expectations of $| {P}_{\Omega }^{{\rm{L}}}|$ and $| -{P}_{\Omega }^{{\rm{L}}}|$ are virtually equal.

In addition, in Fig. 2b, we have show the conditional root-mean-square (rms) of the fluctuations of the ${P}_{\Omega }^{{\rm{L}}}$, normalized in the same manner as Fig. 2a. Once again, we have included the corresponding curve for *η*^2^∇^2^*S*_*ij*_ for comparison. Remarkably, we observe the same behavior as seen in panel Fig. 2a (except the curves are all on the positive side, because the rms is always positive by definition). At the same time, we note that the curves in both Fig. 2a and b, have comparable values, i.e., the mean and rms are comparable (especially for large Ω). This reaffirms that ${P}_{\Omega }^{{\rm{L}}}$ is predominantly negative when conditioned on large values of Ω, and thus consolidates the observed self-attenuation mechanism. Finally, it is worth noting that as *R*/*η* becomes smaller the curves for a given Reynolds number expectedly approach the analytical limit given by Eq. (7). The result for *R*/*η* = 0.5 (not shown), was found to be virtually indistinguishable from the corresponding curve showing the analytical limit.

The observation from Figs. 1 and 2 that extreme vorticity fluctuations are accompanied by negative values of ${P}_{\Omega }^{{\rm{L}}}$ indicates that the strain induced locally acts to prevent further growth of enstrophy. It is important to realize that this mechanism is separate from viscous diffusion or dissipation of enstrophy^15^, but still acts in conjunction with it. In addition, this self-attenuating mechanism is far stronger than a mere reduction (depletion) of non-linearity^19,24^. Depletion of non-linearity essentially refers to weakening of vortex stretching (compared with its maximum possible amplitude)^25^, which is evidently reflected in alignment of vorticity with intermediate eigenvector of strain tensor and hence the weak curvature of vortex tubes^13,26^—as also seen in Fig. 1a–b. However, the presence of self-attenuation suggests that the non-linearity itself could be capable of preventing a runaway blow-up, even as viscosity gets small (as suggested by Fig. 2a, where the increase of ${P}_{\Omega }^{{\rm{L}}}$ is merely shifted to larger values of Ω as viscosity decreases). A careful mathematical analysis of this mechanism and determining mathematical bounds on ${P}_{\Omega }^{{\rm{L}}}$ could possibly reveal a path in establishing global regularity of INSE.

### Connection to helicity

The presence of negative local stretching accompanying intense vorticity raises additional questions about the local flow structure. Given that intense vorticity is arranged in tubes with weak curvature, additional insight could be obtained by a simple kinematic analysis of stretching generated by such structures. To this end, we consider a simple axisymmetric vortex tube with a radius of curvature *R*_*c*_^27–29^. Utilizing a curvilinear polar coordinate system: ($\hat{{\bf{r}}}$, $\hat{{\boldsymbol{\theta }}}$, $\hat{{\bf{s}}}$), which, respectively, correspond to unit vectors in the radial direction, the azimuthal direction and the direction tangent along the (curved) axis of the tube, we assume that the vorticity is of the form ${\boldsymbol{\omega }}={\omega }_{s}(r,s)\ \hat{{\boldsymbol{s}}}+{\omega }_{\theta }(r,s)\ \hat{{\boldsymbol{\theta }}}$. The component *ω*_*s*_ corresponds to azimuthal velocity in the tube similar to a two-dimensional Burgers vortex^30^, whereas the component *ω*_*θ*_ comes from axial velocity along the tube. Thereafter, utilizing Eq. (7), one can derive (as shown in the Supplementary Note 2):

8 $${P}_{\Omega }^{{\rm{L}}}(R)=-\frac{{R}^{2}}{10}\left[{\mathcal{F}}\{{\omega }_{s},{\omega }_{\theta }\}+{\mathcal{G}}\{{\omega }_{s},{\omega }_{\theta }\}\frac{\cos \theta }{{R}_{c}}\right]+{\mathcal{O}}({R}^{4})\,,$$

which gives the local stretching induced by the vortex tube as sum of two terms, involving ${\mathcal{F}}$ and ${\mathcal{G}}$, which are functions of *ω*_*s*_ and *ω*_*θ*_ and their derivatives.

The term with a $\cos \theta /{R}_{c}$ dependence results from the curvature of the tube and produces a dipolar structure, with positive and negative contributions depending on the sign of $\cos \theta$^31^—consistent with the structure seen in Fig. 1g and i. In contrast, the term independent of $\cos \theta$ acts as a monopole. Based on the results shown in Figs. 1 and 2, the sign of ${\mathcal{F}}$ must be positive, and would result in attenuation of intense vorticity by the local strain. Interestingly, ${\mathcal{F}}$ is identically zero if the component *ω*_*θ*_ vanishes, i.e., there is no axial flow velocity. This suggests that some local alignment between vorticity and velocity must occur when vorticity is large. In fact, a similar conclusion can also be reached by realizing that the non-linear terms in INSE, in Eq. (1), can be rewritten as  ∇ × (**u** × ***ω***)^19^. Thus, local Beltramization, i.e., alignment of **u** and ***ω*** in regions of large enstrophy would essentially act to restrict the non-linear amplification^19^.

**Fig. 3:**
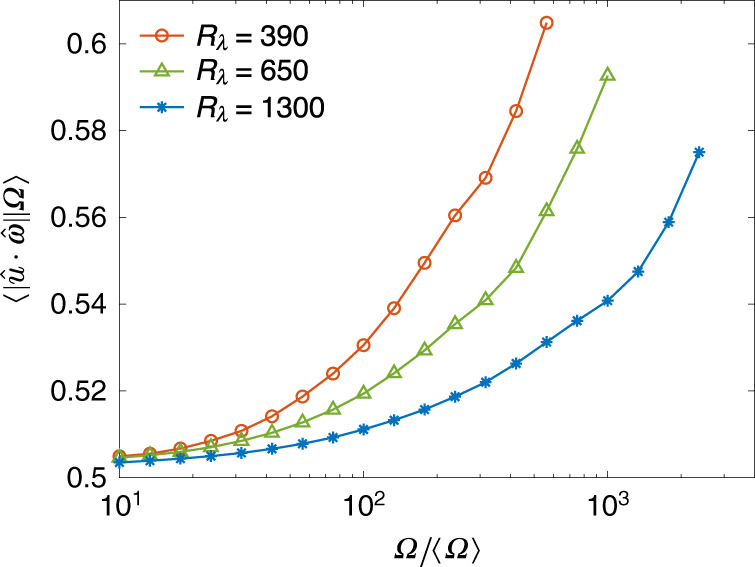
Preferential alignment of vorticity and velocity in regions of intense vorticity. Averaged absolute value of the cosine between velocity and vorticity vectors, conditioned on enstrophy relative to its mean value, at Taylor-scale Reynolds numbers *R*_*λ*_ = 390–1300.

The above prediction is consistent with earlier results at low *R*_*λ*_^32^, as well as with our own results at significantly higher *R*_*λ*_ in Fig. 3, which shows the conditional average of the cosine between velocity and vorticity, conditioned on enstrophy. The average is taken over the absolute value, as the sign of the cosine is immaterial to measure the degree of Beltramization (also note that the dot product of velocity and vorticity is not sign-definite). For small values of Ω, the average stays constant at 0.5, consistent with a uniform distribution of the cosine. However, the conditional average increases at large Ω, in good correlation with the increase of the magnitude of ${P}_{\Omega }^{{\rm{L}}}$ seen in Fig. 2a. Thus, in fully developed turbulence, the intense whirling motions (vortex tubes), emblematic of the small-scale structures, are innately three-dimensional and helical.

## Discussion

We have utilized very well-resolved numerical simulations of fully developed turbulence to investigate extreme fluctuations of vorticity, which can be considered as signatures of potential singularities of INSE. Our results show that when vorticity is strongly amplified, the non-linearity in its local neighborhood remarkably counteracts further amplification, instead of enhancing it. In addition, this effect gets stronger as vorticity gets stronger and also as Reynolds number increases (or viscosity decreases). Thus, our results suggest that the non-linearity, which is responsible for amplification in the first place, encodes a mechanism (in conjunction with viscosity), which can prevent a finite-time singularity from occurring. A deeper understanding of this self-attenuation mechanism based on a clear physical argument could help to set stronger mathematical bounds on the amplification of vorticity^18,33^, and could be an essential ingredient to prove global regularity of the INSE^3^.

Another important observation in this regard is the local Beltramization of the flow in regions of large enstrophy—highlighting the helical nature of the small scales of turbulence (which are structurally arranged in vortex tubes). Although it was anticipated that reduction (depletion) of non-linearity would lead to such helicity^19^, the uncovered self-attenuation mechanism shows that the effect is in fact much stronger, and directly counteracts vorticity amplification. A promising direction in this regard could be to extend the ideas based on helical decomposition to further analyze this local Beltramization^34,35^. Indeed, a recent work has established global regularity for a decimated version of INSE, which enforces helicity to be sign-definite^36^. A possible extension to full INSE, in light of the uncovered self-attenuation mechanism, presents an important challenge for future work.

On a related note, it is worth mentioning that our numerical simulations of stationary isotropic turbulence do not address specific initial value problems, such as those involving collisions between two or more vortex tubes^31,37–39^. Such special flow configurations are routinely studied to investigate the development of a possible finite-time singularity, mostly in the context of inviscid flows (*ν* = 0), i.e., the Euler equations. However, a robust demonstration of a blow-up or lack thereof still remains elusive^40^. Athough complicated interactions between vortex tubes already occur in our simulations, it remains to be understood how the ideas developed here would apply to these special configurations.

In conclusion, our analysis of very well-resolved turbulence simulations reveals a novel mechanism encoded in the non-linearity of Navier–Stokes equations, which contrary to expectations, attenuates vorticity amplification in regions where vorticity is most intense (instead of enhancing it). This observation provides important insights on the nature of extreme events in turbulent flows, and in the process also suggests a new way to address the fundamental question whether amplification of vorticity can develop into finite-time singularities.

## Methods

### Description of DNS

The data utilized in the current work are generated through DNS of the INSE

9 $$\partial {\bf{u}}/\partial t+{\bf{u}}\cdot \nabla {\bf{u}}=-\nabla P/\rho +\nu {\nabla }^{2}{\bf{u}}+{\bf{f}}\,,$$

where **u** is the divergence free velocity field ( ∇ ⋅ **u** = 0), *P* is the pressure, *ρ* is the fluid density, *ν* is the kinematic viscosity, and **f** corresponds to large scale forcing used to maintain a statistically stationary state^41^. The equations are solved using a massively parallelized version of the well-known Fourier pseudo-spectral algorithm of Rogallo^42^. The aliasing errors resulting from the convolution sums are controlled by grid shifting and spherical truncation^43^. Our DNS corresponds to the canonical setup of homogeneous and isotropic turbulence with periodic boundary conditions on a cubic domain of side length *L*_0_ = 2*π*, which is ideal for studying small scales and hence extreme events at highest Reynolds numbers possible^11^. The domain is discretized using *N*^3^ grid points, with uniform grid spacing Δ*x* = *L*_0_/*N* in each direction. We utilize explicit second-order Runge–Kutta for time integration, where the time step Δ*t* is subject to the Courant number (*C*) constraint for numerical stability: Δ*t* = *C*Δ*x*/∣∣**u**∣∣_*∞*_ (where ∣∣ ⋅ ∣∣_*∞*_ is the *L*^*∞*^ norm).

The DNS database used in the current work is summarized in Table 1, along with the main simulation parameters. An important consideration in studying extreme events is that of spatial resolution, which is measured in pseudo-spectral DNS by the parameter ${k}_{\max }\eta$, where ${k}_{\max }=\sqrt{2}N/3$ is the maximum resolved wavenumber on a *N*^3^ grid and *η* is the Kolmogorov length scale. Equivalently, one can use the ratio Δ*x*/*η* which is approximately equal to $3/{k}_{\max }\eta$. The runs with Taylor-scale Reynolds numbers, *R*_*λ*_, in the range 140 ≤ *R*_*λ*_ ≤ 650 were also utilized in our recent work^12^ and all have a very high spatial resolution, ${k}_{\max }\eta \approx 6$ (or Δ*x*/*η* ≈ 0.5). This resolution should be compared with the one used in comparable numerical investigations of turbulence at high Reynolds numbers, which are mostly in the range $1\le {k}_{\max }\eta \le 1.5$^11,44^—which do not resolve the extreme events adequately. In addition to our previous runs, we have performed a new run at significantly higher *R*_*λ*_ of 1300, on a larger 12288^3^ grid with a small-scale resolution of ${k}_{\max }\eta =3$ (or Δ*x*/*η* ≈ 1). This is one of the largest DNS reported to date—comparable with^21^, which also reported results from 12288^3^ run at *R*_*λ*_ = 2300, but with ${k}_{\max }\eta \approx 1$ (where the small scales were not properly resolved).

**Table 1:** Simulation parameters for the DNS runs used in the current work: the Taylor-scale Reynolds number (*R*_*λ*_), the number of grid points (*N*^3^), spatial resolution (${k}_{\max }\eta$), ratio of large-eddy turnover time (*T*_E_) to Kolmogorov time scale (*τ*_K_), length of simulation (${T}_{{\rm{sim}}}$) in statistically stationary state, and the number of instantaneous snapshots (*N*_s_) used for each run to obtain the statistics.

*R*_*λ*_	*N*^3^	${k}_{\max }\eta$	*T*_E_/*τ*_K_	${T}_{{\rm{sim}}}$	*N*_s_
140	1024^3^	5.82	16.0	6.5*T*_E_	24
240	2048^3^	5.70	30.3	6.0*T*_E_	24
390	4096^3^	5.81	48.4	4.0*T*_E_	35
650	8192^3^	5.65	74.4	2.0*T*_E_	40
1300	12288^3^	2.95	147.4	20*τ*_K_	18

We have also listed the simulation length ${T}_{{\rm{sim}}}$ used for generating independent ensembles, in terms of the large-eddy turnover time (*T*_E_) or the Kolmogorov time scale (*τ*_K_). The statistical results are obtained by averaging over *N*_s_ independent ensembles, which are uniformly spread out over the simulation length. Note, the range of time scales is typically given by the ratio *T*_E_/*τ*_K_, which scales linearly with *R*_*λ*_^1^. However, the time scale of extreme events, which we consider here is smaller than *τ*_K_, getting even smaller as *R*_*λ*_ increases^12^.

## Supplementary information

Supplementary Information

## Data Availability

The data that support the findings of this study are available from the corresponding author on request.

## Code availability

The simulation and post-processing codes that have been used to produce the results of this study are available from the corresponding author on request.
