# Supplementary material for: Self-attenuation of extreme events in Navier–Stokes turbulence
Source: Nat Commun. 2020 Nov 17;11:5852. doi: 10.1038/s41467-020-19530-1 (PMC7672113; doi:10.1038/s41467-020-19530-1)
Supplement: Supplementary file 1 — Supplementary Information [file 41467_2020_19530_MOESM1_ESM.pdf]

Supplementary Information
Self-attenuation of extreme events in Navier-Stokes turbulence

Dhawal Buaria, Alain Pumir and Eberhard Bodenschatz

SUPPLEMENTARY FIGURE 1

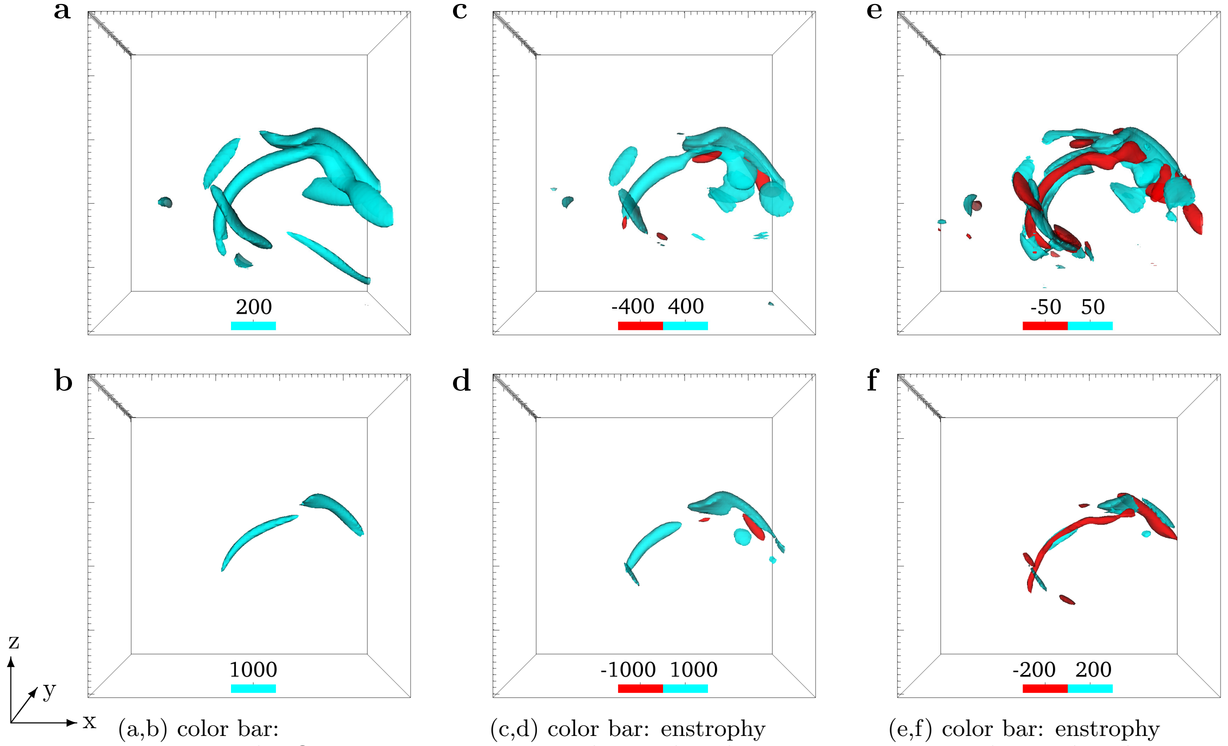

(a,b) color bar: enstrophy, $\Omega = \omega_i\omega_i$

(c,d) color bar: enstrophy production based on total strain, $P_\Omega = \omega_i\omega_j S_{ij}$

(e,f) color bar: enstrophy production based on local strain, $P_\Omega^{\mathrm{L}} = \omega_i\omega_j S_{ij}^{\mathrm{L}}$

SUPPLEMENTARY FIG. 1. **Prevalence of negative local stretching in regions of intense vorticity**. In the same spirit as in Fig.1 of the main text, this figure shows a visualization of the flow around another extreme event. The panels focus on a representative region of intense vorticity from our numerical simulation at Taylor-scale Reynolds number $R_\lambda = 650$ on a 8192^3 grid or equivalently of size $(4096\eta)^3$, where η is the Kolmogorov length scale where viscosity acts. The maximum enstrophy (vorticity-squared) is at the center of the domain shown, whose edges are 50η in each direction (in each panel successive major ticks are 10η apart). Left column: Isosurfaces of enstrophy at thresholds of (a) 200, and (b) 1000 (times the mean value). The structure of the flow turns out to be more complicated than in Fig.1 of the main text, but as the threshold is increased, panel b reveals a simpler underlying structure of isolated tubes. Middle column: enstrophy production based on total strain, suitably non-dimensionalized by mean of enstrophy, at thresholds of (c) ± 400, and (d) ± 1000, which approximately correspond to moderate and intense enstrophy, shown in (a) and (b) respectively. The production based on total strain is overwhelmingly positive. Right column: enstrophy production based on local strain (for $R = 2\eta$), once again suitably non-dimensionalized by mean enstrophy, at thresholds of (e) ± 50, and (f) ± 200, again corresponding to moderate and intense enstrophy shown in (a) and (b) respectively.

SUPPLEMENTARY NOTE 1: DETERMINATION OF THE NON-LOCAL STRAIN

We start with Eq. 8 of the main text, which expresses the non-local strain in terms of the total strain:

$$S_{ij}^{\mathrm{NL}}(\mathbf{x},R) = \left[1 + \frac{R^2}{10}\nabla^2 + \frac{R^4}{280}\nabla^2\nabla^2 + ... + \frac{3R^{2n-2}}{(2n-2)!(4n^2-1)}(\nabla^2)^{n-1} + ...\right] S_{ij}(\mathbf{x}) \ . \tag{1}$$

In order to evaluate the above expression, we apply a Fourier transform to both sides. Denoting $\mathbf{k}$ as the wavenumber vector and $k = |\mathbf{k}|$, the operator ∇^2 reduces to a multiplication by $-k^2$ in Fourier space [1], and the expression becomes:

$$\hat{S}_{ij}^{\mathrm{NL}}(\mathbf{k},R) = f(kR)\ \hat{S}_{ij}(\mathbf{k}) \ , \tag{2}$$

where $\hat{(\cdot)}$ denotes the Fourier transform and $f(kR)$ is an infinite series given as

$$f(kR) = 1 - \frac{(kR)^2}{10} + \frac{(kR)^4}{280} + ... + \frac{3(-1)^{n-1}(kR)^{2n-2}}{(2n-2)!(4n^2-1)} + ... \tag{3}$$

It can be readily seen that the radius of convergence of this series is infinite. To show that Eq. (3) reduces to an analytical expression, we rewrite the series in a compact form by setting $kR = x$:

$$f(x) = \sum_{n=1}^{\infty} \frac{3(-1)^{n-1}\ x^{2n-2}}{(2n-2)!(4n^2-1)} \ . \tag{4}$$

By factoring $4n^2 - 1 = (2n-1)(2n+1)$ and multiplying the numerator and denominator by $2n$, the above expression can be rewritten as

$$f(x) = \sum_{n=1}^{\infty} \frac{3(-1)^{n-1}\ (2n)\ x^{2n-2}}{(2n+1)!} \ . \tag{5}$$

Now by multiplying both sides by x, we get:

$$xf(x) = \sum_{n=1}^{\infty} \frac{3(-1)^{n-1}\ (2n)\ x^{2n-1}}{(2n+1)!} \ , \tag{6}$$

and thereafter the factor $2n$ in the numerator can be absorbed by writing the right-hand side as a derivative in the following form:

$$xf(x) = \frac{d}{dx}\left(\sum_{n=1}^{\infty} \frac{3(-1)^{n-1}\ x^{2n}}{(2n+1)!}\right) \ . \tag{7}$$

In this step, by noting that the expression inside the summation is a constant independent of x for $n = 0$, we can simply adjust the limits of summations to start from $n = 0$ to obtain

$$xf(x) = \frac{d}{dx}\left(\sum_{n=0}^{\infty} \frac{3(-1)^{n-1}\ x^{2n}}{(2n+1)!}\right) \ . \tag{8}$$

Finally, we can take out a factor of $-3/x$ from the summation (since it does not depend on n) to get

$$xf(x) = \frac{d}{dx}\left(-\frac{3}{x}\sum_{n=0}^{\infty} \frac{(-1)^{n}\ x^{2n+1}}{(2n+1)!}\right) \ , \tag{9}$$

where the summation term is exactly the power series expansion for $\sin x$, giving

$$xf(x) = \frac{d}{dx}\left(\frac{-3\sin x}{x}\right) \; , \tag{10}$$

which after simplification gives the desired result

$$f(x) = \frac{3\left[\sin x - x\cos x\right]}{x^3} \; . \tag{11}$$

Interestingly, this is the sinc function in 3D, which also happens to be the Fourier transform of the box or top-hat filter (or the indicator function of the ball of radius R [2]), and shows up in many other scientific contexts. Thus, obtaining the non-local strain essentially reduces to a filtering operation on the total strain.

SUPPLEMENTARY NOTE 2: LOCAL STRETCHING NEAR A VORTEX TUBE

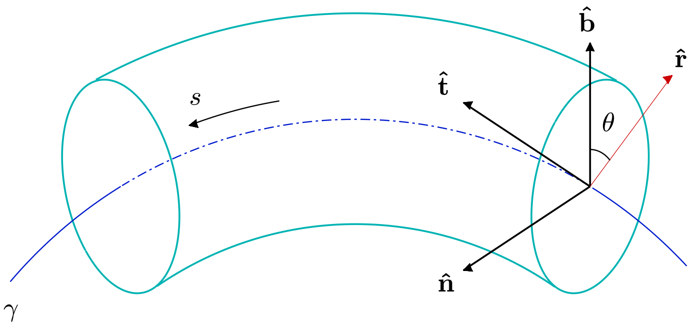

SUPPLEMENTARY FIG. 2. **Coordinate system along the vortex tube**. γ is the curve along the tube axis and s is the curvilinear distance measured along the axis. ($\hat{\mathbf{t}}$, $\hat{\mathbf{n}}$, $\hat{\mathbf{b}}$) is the Frenet-Serret frame, and (r, θ, s) is the curvilinear cylindrical coordinate system (note $\hat{\mathbf{s}} = \hat{\mathbf{t}}$).

In this section we analyze the straining field generated locally in the vicinity of a slender vortex filament. We determine the stretching term, $P_\Omega^{\mathrm{L}} = \omega_i \omega_j S_{ij}^{\mathrm{L}}$, and explain, the structure observed numerically in Fig.1 of the main text.

As shown in Supplementary Fig. 2, consider a vortex tube localized around a curve, γ, parametrized by arclength, s. It is convenient to introduce a system of curvilinear coordinates [3]. To this end, we begin with the classical Frenet-Serret frame, consisting of $\hat{\mathbf{t}}$, $\hat{\mathbf{n}}$ and $\hat{\mathbf{b}}$, respectively the unit vectors tangent, normal and binormal to the curve (the unit vector $\hat{\mathbf{s}}$ along the curve is simply $\hat{\mathbf{t}}$). Close to a point $P(s)$ located on the curve γ, we parametrize a position M in the plane, normal to the tangent vector $\hat{\mathbf{t}}$, by a polar coordinate system, (r, θ), the angle being measured from the position of $\hat{\mathbf{b}}$. The set of coordinates (r, θ, s) provides a parametrization of the position around γ, within a distance of $\mathcal{O}(R_\mathrm{c})$ of γ, where R_c is the radius of curvature of γ. Varying $(r, \theta, s) \to (r + dr, \theta + d\theta, s + ds)$ leads to the expression for an infinitesimal displacement:

$$d\mathbf{M} = dr\ \hat{\mathbf{r}} + r d\theta\ \hat{\boldsymbol{\theta}} + h_s ds\ \hat{\mathbf{s}} \tag{12}$$

where

$$\hat{\mathbf{r}} = \cos\theta\ \hat{\mathbf{b}} - \sin\theta\ \hat{\mathbf{n}}\ , \tag{13}$$

$$\hat{\boldsymbol{\theta}} = -\sin\theta\ \hat{\mathbf{b}} - \cos\theta\ \hat{\mathbf{n}}\ , \tag{14}$$

and h_s is given as:

$$h_s = \left(1 + \frac{r}{R_\mathrm{c}} \sin\theta\right)\ . \tag{15}$$

Here, R_c the radius of curvature of the curve γ.

We consider a vorticity field localized close to γ, which we write as:

$$\boldsymbol{\omega} = \omega_s(r, s)\, \hat{\mathbf{s}} + \omega_\theta(r, s) \hat{\boldsymbol{\theta}} \tag{16}$$

In the case of a straight vortex tube ($R_\mathrm{c} \to \infty$), the component parallel to $\hat{\mathbf{s}}$ is responsible for the azimuthal component of velocity, whereas the component ω_θ is associated with a vertical component of velocity:

$$u_\theta(r, s) = \frac{1}{r} \int_0^r r' \omega_s(r', s) dr' \qquad \text{and} \qquad u_z(r, s) = -\int_r^\infty \omega_\theta(r', s) dr' \tag{17}$$

The axial vorticity component ω_s characterises an axisymmetric Burgers-like vortex [4] with a purely 2D structure and has been utilized in many contexts in the literature. However, as shown soon, the component ω_θ is also necessary to explain the structure shown in Fig.1 of the main text (which cannot result from a purely 2D vortex tube).

To estimate the local stretching $P_\Omega^{\rm L}$ at a point close to the vortex tube, we use the approximation, deduced from Eq.(5) of the main text, and reformulated by using the well-known identity $\nabla\times\boldsymbol{\omega} = -\nabla^2\mathbf{u}$:

$$S_{ij}^{\rm L} = \frac{R^2}{20}\Big[\partial_i\zeta_j + \partial_j\zeta_i\Big] \quad \text{with} \quad \boldsymbol{\zeta} = \nabla\times\boldsymbol{\omega} \tag{18}$$

To compute $\nabla\times\boldsymbol{\omega}$, we use the general formula for the rotational in any curvilinear system [5]:

$$\nabla\times\boldsymbol{\omega} = \frac{\hat{\mathbf{r}}}{rh_s}[\partial_\theta(h_s\omega_s) - \partial_s(r\omega_\theta)] - \frac{\hat{\boldsymbol{\theta}}}{h_s}\partial_r(h_s\omega_s) + \frac{\hat{\mathbf{s}}}{r}\partial_r(r\omega_\theta) \tag{19}$$

We consider the case of a weakly curved vortex tube, with weak variations along the axis of the vortex. In this case, $1/R_{\rm c} = 0$, $h_s \to 1$ and $\partial_s \to 0$, so Eq. (19) reduces to:

$$\nabla\times\boldsymbol{\omega} = -\hat{\boldsymbol{\theta}}\;\partial_r\omega_s + \hat{\mathbf{s}}\;\frac{1}{r}\partial_r(r\omega_\theta) \tag{20}$$

In the case where both $1/R_{\rm c}$ and ∂_s are very small compared to the size of the vortex and to the partial derivative ∂_r, we re-organize the expression for $\nabla\times\boldsymbol{\omega}$, Eq. (19), as a sum of the dominant term, due to the almost straight vortex tube, Eq. (20), plus a small perturbation generated by the variations of the quantities along the filament (∂_s) and by curvature ($1/R_{\rm c}$). Here, we keep only the lowest order terms in a formal expansion in powers of $1/R_{\rm c}$ and ∂_s. This leads to the following expression, in the orthonormal basis $(\hat{\mathbf{r}}, \hat{\boldsymbol{\theta}}, \hat{\mathbf{s}})$:

$$\nabla\times\boldsymbol{\omega} = \begin{pmatrix} 0 \\ -\partial_r\omega_s \\ \frac{1}{r}\partial_r(r\omega_\theta)\end{pmatrix} + \begin{pmatrix} (\frac{\cos\theta}{R_{\rm c}}\omega_s - \partial_s\omega_\theta) \\ -\frac{\sin(\theta)}{R_{\rm c}}\omega_s \\ 0\end{pmatrix} \equiv \begin{pmatrix}\zeta_r \\ \zeta_\theta \\ \zeta_s\end{pmatrix} \tag{21}$$

Interpreting $\boldsymbol{\zeta}$ as a velocity, Eq. (18) effectively reduces to a rate-of-strain tensor, $e_{\alpha\beta}$, whose components are [5]:

$$e_{\theta\theta} = \frac{1}{r}\partial_\theta\zeta_\theta + \frac{\zeta_r}{r} \approx -\frac{1}{r}\partial_s\omega_\theta \tag{22}$$

$$e_{ss} = \frac{1}{h_s}\partial_s\zeta_s + \zeta_r\frac{\sin(\theta)}{R_{\rm c}} + \zeta_\theta\frac{\cos\theta}{R_{\rm c}} \approx \frac{1}{r}\partial^2_{sr}(r\omega_\theta) - \frac{\cos\theta}{R_{\rm c}}\partial_r\omega_s \tag{23}$$

$$e_{s\theta} = \frac{r}{2h_s}\partial_s(\frac{\zeta_\theta}{r}) + \frac{h_s}{2r}\partial_\theta(\frac{\zeta_s}{h_s}) \approx -\frac{1}{2}\partial^2_{sr}\omega_s - \frac{\cos\theta}{2rR_{\rm c}}\partial_r(r\omega_\theta) \tag{24}$$

The approximate expressions of e, on the right-hand-side of Eqs. (22-24) were obtained by keeping only the lowest order terms in a formal expansion, based on the small values of ∂_s and $1/R_{\rm c}$. Thereafter, the expression for $P_\Omega^{\rm L}$ can be written as:

$$\begin{aligned} P_\Omega^{\rm L} &\approx \frac{R^2}{20}\Big[\frac{1}{3r}\partial_s(\omega_\theta^3) + \frac{1}{r}\partial^2_{sr}(r\omega_\theta)\omega_s^2 - \frac{\cos\theta}{3R_{\rm c}}\partial_r(\omega_s^3) - \{\partial^2_{sr}(\omega_s) + \frac{\cos\theta}{rR_{\rm c}}\partial_r(r\omega_\theta)\}\omega_s\omega_\theta\Big] \\ &\approx \frac{R^2}{20}\Big[\frac{1}{3r}\partial_s(\omega_\theta^3) + \frac{1}{r}\partial^2_{sr}(r\omega_\theta)\omega_s^2 - \partial^2_{sr}(\omega_s)\omega_s\omega_\theta\Big] \\ &\quad - \frac{R^2}{20}\Big[\frac{1}{3}\partial_r(\omega_s^3) + \frac{1}{r}\partial_r(r\omega_\theta)\omega_s\omega_\theta\Big]\frac{\cos\theta}{R_{\rm c}} \end{aligned} \tag{25}$$

The expression Eq. (25) for P_Ω^{L} appears as a sum of two terms with different symmetries, as stated in Eq.(6) of the main text. In fact, comparing Eq. (25) with Eq.(6) of the main text leads to:

$$\mathcal{F}\{\omega_s, \omega_\theta\} = -\Big[\frac{1}{3r}\partial_s(\omega_\theta^2) + \frac{1}{r}\partial_{sr}^2(r\omega_\theta)\omega_s^2 - \partial_{sr}^2(\omega_s)\omega_s\omega_\theta\Big] \tag{26}$$

$$\mathcal{G}\{\omega_s, \omega_\theta\} = \Big[\frac{1}{3}\partial_r(\omega_s^3) + \frac{1}{r}\partial_r(r\omega_\theta)\omega_s\omega_\theta\Big] \tag{27}$$

The term proportional to $\cos\theta$, with a dipolar symmetry, is proportional to $1/R_{\mathrm{c}}$ and therefore induced by the curvature of the vortex only. The presence of this term is clearly visible in Fig.1g of the main manuscript, and can be explained from an elementary Biot-Savart calculations of the velocity field from a curved vortex tube [6]. The dipolar term changes sign under the transformation $\omega_s \to -\omega_s$, which corresponds in the Biot-Savart expression to flipping the sign of the circulation. The monopolar term, independent of θ, is the source of the strong negative P_Ω^{L} contribution, clearly seen in Fig.1. This term is uniformly 0 when $\omega_\theta = 0$, which implies that the monopolar contribution to P_Ω^{L} is entirely due to the axial velocity in the tube.

SUPPLEMENTARY REFERENCES

[1] S. B. Pope, *Turbulent Flows* (Cambridge University Press, 2000).

[2] I. S. Gradzhteyn and I. M. Ryzhik, *Tables of integrals, series and products*, seventh edition ed. (Elsevier Inc., 2007).

[3] A. Pumir and E. D. Siggia, "Vortex dynamics and the existence of solutions to the Navier-Stokes equations," Phys. Fluids **30**, 1606–1626 (1987).

[4] J. M. Burgers, "A mathematical model illustrating the theory of turbulence," Adv. Appl. Mech. **1**, 171–99 (1948).

[5] G. K. Batchelor, *An introduction to Fluid Dynamics* (Cambridge University Press, Cambridge, 1967).

[6] A. Pumir and E. D. Siggia, "Collapsing solutions to the 3D Euler equations," Phys. Fluids A **2**, 220–241 (1990).
